# Drug-free in vitro activation combined with 3D-bioprinted adipose-derived stem cells restores ovarian function of rats with premature ovarian insufficiency

**DOI:** 10.1186/s13287-022-03035-3

**Published:** 2022-07-26

**Authors:** Qian Li, Jiahua Zheng, Zhongkang Li, Yanlai Xiao, Mingle Zhang, Wenxin Shi, He Gao, Xianghua Huang, Jingkun Zhang

**Affiliations:** Department of Obstetrics and Gynecology, The Second Hospital of Hebei Medical University, 215 Heping West Road, Shijiazhuang, 050000 Hebei China

**Keywords:** Drug-free in vitro activation, 3D-bioprinting, Adipose-derived stem cells, Decellularized extracellular matrix, Premature ovarian insufficiency, Vascularization, PI3K/AKT signaling pathway

## Abstract

**Background:**

Emerging drug-free in vitro activation (IVA) technique enables patients with premature ovarian insufficiency (POI) to restore ovarian function and conceive their own genetic offspring. However, various issues have greatly restricted its clinical application. Transplantation of adipose-derived stem cells (ADSCs) has promising roles in restoring ovarian function of rats with POI, but insufficient retention has greatly hampered their efficiency. Here, we designed a 3D-bioprinted engineering ovary composed of drug-free IVA and ADSCs, which may prolong the retention of ADSCs and construct an early vascular microenvironment, thus compensating for the disadvantages of drug-free IVA to some extent and ameliorating impaired ovarian function in the POI rats.

**Methods:**

After intraperitoneal injection of cyclophosphamide, the POI model rats were randomized into 5 groups: (1) POI group; (2) ovarian fragments group; (3) 3D scaffold combined with ovarian fragments group; (4) ovarian fragments combined with ADSCs group; (5) 3D scaffold with ADSCs combined with ovarian fragments as 3D-bioprinted engineering ovary group. Normal rats were identified as the control group. The localization of CM-Dil-labeled ADSCs and co-localization with CD31 were observed to examine the distribution and underlying mechanism of differentiation. Histomorphological and immunohistochemical analyses were performed to calculate follicle number and assess proliferation and apoptosis of granulosa cells (GCs). Immunofluorescence staining was used to evaluate angiogenesis. Hormone levels were measured to evaluate the restoration of endocrine axis. Western blot analysis and RT-PCR were conducted to explore the potential mechanism.

**Results:**

CM-Dil-labeled ADSCs were distributed in the interstitium of ovaries and had significantly higher retention in the 3D-bioprinted engineering ovary group. Several regions of the co-staining for CM-Dil and CD31 were in the area of vascular endothelial cells. Meanwhile, the follicle counts, GCs proliferation, neoangiogenesis, and hormone levels were significantly improved in the 3D-bioprinted engineering ovary group, as compared with other groups. Furthermore, the ovarian function was ameliorated and angiogenesis was promoted through regulating the PI3K/AKT pathway.

**Conclusion:**

Our results suggested that 3D-bioprinted engineering ovary had great potential for restoring impaired ovarian function of rats with POI, which could compensate for the disadvantages of drug-free IVA to some extent.

**Supplementary Information:**

The online version contains supplementary material available at 10.1186/s13287-022-03035-3.

## Background

Premature ovarian insufficiency (POI) that occurs in 1% of women younger than 40 years would ultimately be the clinical manifestation of accelerated ovarian follicular exhaustion, for which the only option is egg donation [1]. Despite residual dormant follicles in several POI patients, they are less likely to grow after traditional treatment and difficult to fertilize with their own eggs. In vitro activation (IVA) of residual dormant follicles is emerging as a novel effective approach for restoring fertility to women with POI, which enables them to conceive their own genetic offspring [2, 3]. This approach is established based on the ovarian fragmentation which activates resting follicles by disrupting Hippo signaling pathway and on manipulation of the phosphatase and tensin homolog (PTEN)/phosphatidylinositol-3-kinase (PI3K)/protein kinase B (AKT)/forkhead box O3 (FOXO3) pathway, which activates primordial follicles [2, 3]. However, due to the low rate of pregnancy and potential carcinogenic effects of pharmacologic approaches, a simplified drug-free IVA method to promote follicle growth by disrupting Hippo signaling pathway alone was put forward [4, 5]. To date, several success pregnancies and live births have been reported after the IVA [6–12]. Compared with conventional IVA, researchers have assumed that the drug-free IVA seemed to be more efficient [13]. Despite ample evidence that supports the effectiveness of drug-free IVA, there are still numerous issues that have greatly restricted its application, such as low pregnancy rates, loss of a substantial number of primordial follicles, and unimproved quality of age-associated oocytes [2, 13].

To our knowledge, ischemic injury from delayed revascularization between ovarian tissues and recipients is a major cause of premature follicle depletion and low survival of grafts [14]. Therefore, the future studies should concentrate on promoting early post-transplantation revascularization and improving fertility associated with age-related declines in oocyte quality. Numerous studies have demonstrated that mesenchymal stem cells (MSCs) could significantly stimulate neovascularization and increase blood perfusion to reduce the loss of primordial follicles [15, 16] and effectively improve the quality and number of oocytes in aged mice [17–19]. This represents a new strategy toward boosting fertility in women with reduced ovarian reserve. Adipose-derived stem cells (ADSCs), a type of widely used multi-potent stem cells, are ideal seed cells, which possess the characteristics of easily access, great proliferation and low immunogenicity [20]. Although ADSCs have shown potency in recovering ovarian function and enhancing fertility, quick diffusion and insufficient retention are the major restrictions for the application of ADSCs therapy. Therefore, various types of scaffolds encapsulating ADSCs have been applied to increase both short- and long-term retention to further enhance the efficiency of ADSCs [21, 22].

Decellularized extracellular matrix (dECM), which is derived from natural tissues, has been treated to maximally remove cellular and antigenic components to minimize immune response, while preserving the natural skeleton of ECM [23]. The dECM-based scaffold recreates excellent in vivo ovarian microenvironment, ensuring the MSCs and ovarian cell viability, facilitating the cells for necessary interaction with their surrounding environment [24], and also supporting the viability and function of in vitro cultured ovarian cortical tissues [25]. Therefore, we focused on utilizing 3D-bioprinted scaffold based on ovarian dECM-based “bioinks” encapsulating ADSCs, supporting more cell retention and survival in the target tissue, as well as exhibiting a powerful pro-angiogenesis effect, which in turn reduces ischemic injury of the follicles.

Given the potential role of the early revascular microenvironment constructed by 3D-bioprinted ovarian scaffold, as well as IVA of residual dormant follicles, we hypothesized that the combination of aforementioned two approaches could be applied to minimize ischemic injury of ovarian tissue through enhancing early angiogenesis and blood oxygen supply, thus restoring ovarian function. Based on the above hypothesis, our research was designed for a novel 3D-bioprinted engineering ovary and assessed the effect of the above system on the revascularization in the grafts and restoration of ovarian function in the POI rats.

## Methods

### ADSCs culture and characterization

The isolation and cultivation of ADSCs were performed according to previously published protocol [26]. Briefly, inguinal adipose tissues were collected from female Sprague–Dawley rats at the age of 3–4 weeks. The tissues were washed extensively with sterile phosphate-buffered saline (PBS; Servicebio, China), and each adipose segment was chopped with scissors and enzymatically digested with 0.1% type I collagenase (Invitrogen, USA) at 37 °C for 30 min with gentle agitation. Subsequently, the cellular precipitate was obtained by centrifugation at 1000*g* for 10 min. Then, the precipitate was re-suspended twice in PBS and cultured in serum-free basic MSC medium (Beijing Jing-Meng Cell Biological Technology Co., Ltd., China) supplemented with serum-free nutritional substance, and 1% penicillin/streptomycin (Gibco, USA) in a humidified incubator with 5% carbon dioxide at 37 °C. After approximately every 48 h, the culture medium was changed and passaged until cells reached 90% confluence.

The specific surface antigens of ADSCs (Passage 3–5) such as CD45, CD90, CD105, CD29, CD73, CD44 and HLA-DR (Biosciences, USA) were identified by flow cytometry (FCM). The multi-potency of ADSCs was verified by osteogenic and adipogenic differentiation following the manufacture’s protocol. In brief, ADSCs of 90% confluence were co-incubated with adipogenic induction medium and osteogenic induction medium (Gibco, USA) for up to 14 or 28 d, and induced cells were detected by Oil Red O and Alizarin red (Sigma, USA), respectively.

### Acquisition, decellularization, and histological analysis of porcine ovarian tissue

Porcine ovaries were harvested from a local abattoir (Shijiazhuang, China) from 6-month-old pigs and transferred to the laboratory in precooling saline. Upon arrival at the laboratory, according to the process we described previously [27], redundant or unwanted tissues and ovary medulla were removed. The ovarian cortex was cut into 500-µm small and fusiform strips, and after being rinsed multiple times with water to remove excess plasma, all samples were decellularized, lyophilized and stored in − 20 °C refrigerator. Native and decellularized porcine ovarian tissue samples were fixed in 4% paraformaldehyde, respectively, for 24–48 h at room temperature, followed by hematoxylin and eosin staining (HE; Servicebio, China), Masson staining (Servicebio, China) and 4′6-Diamidino-2-Phenylindole staining (DAPI; Southern Biotech, China).

### 3D “bioinks” preparation and 3D printing

3D “bioinks” preparation was performed as we previously described [28]. Briefly, lyophilized dECMs were grinded into powder with the assistance of liquid N2. 100 milligram (mg) of dECM powder and 60 mg of pepsin (porcine, Sigma, USA) were dissolved in 3 ml of concentrated hydrochloric acid (pH = 2.0), mixed well with a small spoon, shaken at 37 °C and 80 × g for 22–24 h. When the primary glue was completely digested, the pH value of dECM solution was re-adjusted to 7.35–7.45 by 10 M NaOH addition to inactivate pepsin and terminate digestion. The mixture of 300 mg of gelatin (Sigma, USA) and 60 mg of sodium alginate (Sigma, USA) in 2 ml tri-ply distilled water was performed at 55 °C for 30 min, which was then mixed with pH 7.35–7.45 dECM solution to generate dECM “bioinks”. dECM “bioinks” disinfection was performed for 3 cycles of 56 °C for 15 min and 4 °C for 15 min.

The liquid dECM “bioinks” mixed thoroughly with/without above-cultured ADSCs (1 × 10^7^ cells mL^−1^) were loaded into the top of barrel and then placed at 4 °C for 15 min to cool the hydrogel into a gel. Using a 3D-Printer (Bio-Architect®-WS; Hangzhou Regenovo Biotechnology, Ltd.,China), dECM “bioinks” were printed from a 340-um stainless steel cooling sprinkler (set at 20 °C) onto a platform controlled at 4 °C. The extrusion pressure was set according to the ink flow velocity (≈ range from 0.18 to 0.32 kPa), and 3D scaffolds (8 × 8 × 3 mm^3^, a circular porous grid scaffold) were printed at a scanning speed of 6mms^−1^. Immediately after printing, the formed structures were cross-linked for 5 min with 5% calcium chloride solution and then washed with sterilized PBS for three times. Scaffolds were then incubated overnight in fresh medium at 37 °C incubator containing 5% CO_2_.

### Establishment of POI rat model

All animal experiments in this study were performed according to the Ethics Committee of the Second Hospital of Hebei Medical University (2021-AE055). Female Sprague–Dawley rats (8–10 weeks old) were provided by Experimental Animal Center of Hebei Medical University (Shijiazhuang, China). The animal models of POI (8–10 weeks old) were induced by daily intraperitoneal injection of cyclophosphamide (CTX; Sigma, USA) (50 mg/kg on the first day and 8 mg/kg/day for the following 14 consecutive days) [29], while the rats in the control group were intraperitoneally administered equivalent volumes of saline. We evaluated the successful establishment of POI model by measuring serum hormone levels, performing pathological analysis of the obtained ovaries, monitoring vaginal smears, and measuring body weight every day for two weeks after CTX injection.

### Intervention of POI rats with subcutaneous implantation of the grafts

The animals that underwent surgical procedure were always in the diestrus phase. Sixty POI rats with disrupted estrous cycle and weight reduction were enrolled and randomized into five groups as follows (*n* = 12, each group): (1) untreated group as POI group; (2) subcutaneous transplantation of ovarian fragments (1 × 1 × 1 mm^3^) alone group as fragment-alone group; (3) subcutaneous 3D scaffold (8 × 8 × 3 mm^3^) combined with ovarian fragments group as scaffold-fragment group; (4) subcutaneous ovarian fragments combined with ADSCs (6 × 10^6^ ADSCs in 60ul PBS per entire rat) group as fragment-cell group; (5)subcutaneous 3D scaffold with ADSCs (1 × 10^7^ ADSCs in 100ul PBS mixed with 1 ml of “bioinks”, 0.6 ml “bioinks” per entire rat) combined with ovarian fragments group as 3D-bioprinted engineering ovary group. Twelve normal rats without any treatment were identified as the control group.

The experimental treatment groups were treated by making 1-cm bilateral micro-incisions on the lower back, and the ovaries were removed from the top of the uterine horns. The surrounding tissues of whole ovaries collected were carefully separated, and ovarian cortex was cut into fragments of 1 × 1 × 1 mm^3^. The scaffold-fragment/3D-bioprinted engineering ovary was established using 3D scaffold (with or without cells) and POI ovarian fragments by wrapping of the latter into 3D stent strut (schematic representation is shown in Fig. 2A). Then, blunt subcutaneous separations were carried out above the dorsal incision to form two tunnels; the scaffold-fragment/3D-bioprinted engineering ovary/ovarian fragments (with or without cells) were placed at both sides of the back, and then, each incision was sutured in an interrupted fashion with 4–0 absorbable sutures (Shanghai Pudong Jinhuan Medical Products Co., LTD), separately. Then, the samples were collected from four rats in each group after killing at 1, 2, and 4 weeks after treatment for subsequent experiments.

### Tracking of transplanted ADSCs in grafts

To track the localization of transplanted ADSCs, approximately 1 × 10^6^ ADSCs were added with 1 ml of culture medium containing 2 mg CM-Dil (Thermo, USA) and kept at 37 °C for 10 min and 4 °C for 20 min. Subsequently, the labeled cells were rinsed twice with sterile PBS to remove the unbound CM-Dil and suspended in PBS for subcutaneous transplantation with ovarian fragments (6 × 10^6^ cells per rat) or thoroughly mixed with “bioinks” (1 × 10^7^ cells ml^−1^ “bioinks”, 0.6 ml “bioinks” per entire rat) for printing. The bilateral grafts were removed at 1 and 4 weeks after transplantation, fixed with optimal cutting temperature (OCT) compound, cut into 6 µm in thickness using a cryostat (Leica, Germany), incubated with DAPI and subsequently imaged under a fluorescence microscope (Olympus, Japan) to examine the survival and distribution of ADSCs in vivo.

### Analysis of ovarian morphology and follicle counts

The grafts from all the experimental groups were collected at 1, 2, and 4 weeks after transplantation, fixed in 4% paraformaldehyde, and then dehydrated in a gradient ethanol followed by xylene vitrification, paraffin-embedment, and sectioning into 5-μm serial sections. The ovarian morphology was recognized by HE staining and visualized using an optical microscope (Zeiss, Germany). We calculated varying stages of follicles (primordial, primary, secondary and antral follicles) in different sections of each group as previously characterized [30]. Only those containing visual oocytes were calculated to avoid overcounting follicles.

### Immunohistochemistry staining and TUNEL analysis

To observe the effects of proliferation and apoptosis of ovarian follicular cells in each group after treatment, the sections were immune-stained with proliferation marker anti-Ki67 antibody (1:800, Servicebio, China) and apoptosis marker anti-caspase-3 antibody (1:800, Servicebio, China) overnight at 4 °C, followed by biotinylated secondary antibody (ZSGB-Bio, China) for 1 h and staining using DAB staining solution (ZSGB-Bio, China). Nuclei were co-stained with hematoxylin. The statistical analysis of Ki67-positive and caspase-3-positive areas was evaluated from four randomly selected regions of discontinuous three sections of each sample. In addition, a TUNEL assay kit (Servicebio, China) was used to further evaluate the apoptosis of representative areas in each group at 4 weeks after transplantation. The procedure was performed following the manufacturer’s introductions. Apoptotic cells were stained with green, while nuclei were stained blue with DAPI.

### Immunofluorescence (IF) staining

Newly formed endothelial surface marker CD31 and proliferating cell nuclear antigen (PCNA) were detected by IF to assess angiogenesis and proliferation of the grafts. Briefly, the sections were blocked in a solution containing 0.2% TritonX-100 and 3% goat serum for 1.5 h and then incubated with anti-CD31 antibody (1:800, Servicebio, China) and anti-PCNA antibody (1:800, Servicebio, China) diluted in antibody diluent overnight at 4 °C followed by incubation with corresponding fluorescently labeled secondary antibody (1:400, Servicebio, China) at 37 °C for 1.5 h. DAPI was routinely used for nuclei counterstaining. The number of CD31-labeled neovascularization from each sample was calculated based on the five randomly selected regions of three discontinuous sections. Furthermore, we applied double-staining with CM-Dil and CD31 to analyze the differentiation of ADSCs in 3D-bioprinted engineering ovary group, and the fresh-frozen sections were obtained from the samples at 4 weeks after transplantation. The specific staining method was performed as described previously. Fluorescent images were acquired using an optical microscope (Zeiss, Germany).

### Hormone assay

At the specified time point of the study (at 1, 2, 4 weeks after transplantation), blood samples were collected from eyeball of the rats in the diestrus and stored overnight at 4 °C, and then, serum was obtained and frozen at − 80 °C for further analysis. Enzyme-linked immunosorbent assay (ELISA) was employed in the measurement of estradiol (E2), follicle-stimulating hormone (FSH), and anti-Müllerian hormone (AMH) according to the manufacturer’s instructions (Cloud-Clone, China). The concentrations of hormone were calculated using standard curves.

### Western blot analysis and real-time PCR (RT-PCR)

Total protein was extracted from ovarian tissues with RIPA lysis buffer (Servicebio, China) supplemented with proteinase inhibitors and phosphatase inhibitors (Servicebio, China). The normalized concentration of proteins was denatured and isolated using 10% SDS-PAGE (Biotides, China) and then transferred to PVDF membranes (Millipore, USA). The membranes were blocked for 1.5 h with 5% bovine serum albumin (BSA; Sigma, USA) (in TBS + 0.1% Tween-20, PH = 7.4), followed by incubation with appropriate primary antibodies as follows: VEGF (1:2000, Zen-bioscience, China), total AKT (1:2000, Zen-bioscience, China), p-AKT (1:1000, Zen-bioscience, China), PI3K (1:1000, Zen-bioscience, China), p-PI3K (1:2000, Zen-bioscience, China) and GAPDH (1:4000, Servicebio, China) at 4 °C overnight. Afterward, the membranes were washed three times and incubated with M5 Goat Anti-Rabbit IgG-HRP (1:10,000, Mei5bio, China) at room temperature for 1 h. Lastly, the protein bands were visualized using the ChemiDoc MP Imaging System (Bio-Rad, USA) and analyzed using Image J software.

Expression levels of angiogenesis-associated mRNA (FGF2, angiogenin, and VEGF) were then quantified by RT-PCR (primer sequences are listed in Table 1). Total RNA was extracted from ovarian tissues using TRIzol Reagent (Servicebio, China) and reverse-transcribed into complementary DNA (cDNA) using the RevertAid First Strand cDNA Synthesis Kit (Thermo, USA). RT-PCR was performed using MonAmp™ ChemoHs qPCR Mix (Monad, China), and the final analysis results were calculated according to 2^−ΔΔCt^ method.

**Table 1:** Primers used for RT-PCR validation

Gene	Forward primer	Reverse primer
VEGF	GCTTTACTGCTGTACCTCCACCATG	CATCTCTCCTATGTGCTGGCTTTGG
Angiogenin	TGGCAACAAGGGCAGCATCAAG	ACTCATCAAAGTGGACAGGCAAGC
FGF-2	CATCACTTCGCTTCCCGCACTG	GCAGCCGTCCATCTTCCTTCATAG
GAPDH	GTCCATGCCATCACTGCCACTC	CGCCTGCTTCACCACCTTCTTG

### Statistical analysis

Statistical analyses of all results were performed using GraphPad Prism 6.0 and SPSS 21.0 software, and the data are expressed as the mean ± SD. F-test was used to assess the homogeneity of variances. A Student *t*-test, one-way ANOVA and Mann–Whitney *U*-test were performed to evaluate the difference between groups. Statistical significance was considered at *P* values < 0.05.

## Results

### Characterization of ADSCs

ADSCs at the third to five passage showed morphologically uniform, adherent, fibroblast-like long spindles. Flow cytometry indicated that the ADSCs were enriched with MSCs’s surface markers CD29 (97.25%), CD73 (90.25%), CD105 (98.09%), CD44 (99.63%), and CD90 (99.66%), but few had positively expressed CD45 (0.24%) and HLA-DR (0.69%). Furthermore, these cells possessed multi-potent characteristics of adipocytes and osteoblasts through in vitro induction, which were concordant with our previous research on ADSCs [31] (Additional file 1: Fig. S1A–C).

### Decellularization of porcine ovarian tissue

We chose porcine ovarian ECM to create a fully functioning engineered ovary because a range of physical and chemical process ensured removal of cellular constituents while sustaining the overall structure and various proteins to support restoration of specific tissue structure and function [32, 33]. To evaluate the validity of decellularization, we conducted histological assays in the native and decellularized tissues. Consequently, we utilized HE staining and DAPI staining to confirm the preservation of cellular components in native tissues and the absence of cellular components in decellularized tissues. Then, Masson staining confirmed that the collagen was well sustained in decellularized tissues (Additional file 2: Fig. S2A). All these results showed that dECM preserved overall structure and composition of native tissue, thereby serving as 3D-bioprinted “bioink” in guiding cell localization, growth, and migration.

### Evaluation of the established POI model

In our study, we observed that body weights of POI group began to drop in the first week after medication, with a marked decline in the second week, which was significantly lower than that in the normal group. Compared with the regular estrous cycle in the normal group, the POI group had a longer diestrus and irregular estrous cycle. In addition, pathological staining revealed that the number of follicles at all levels was remarkably reduced, E2 hormone level was substantially declined, FSH level was significantly increased, and TUNEL staining of apoptotic GCs was obviously increased in the POI group (Additional file 2: Fig. S2B–F). These results indicated that we had successful created a rat model of chemotherapy-induced POI.

### In vivo tracking of ADSCs

**Fig. 1:**
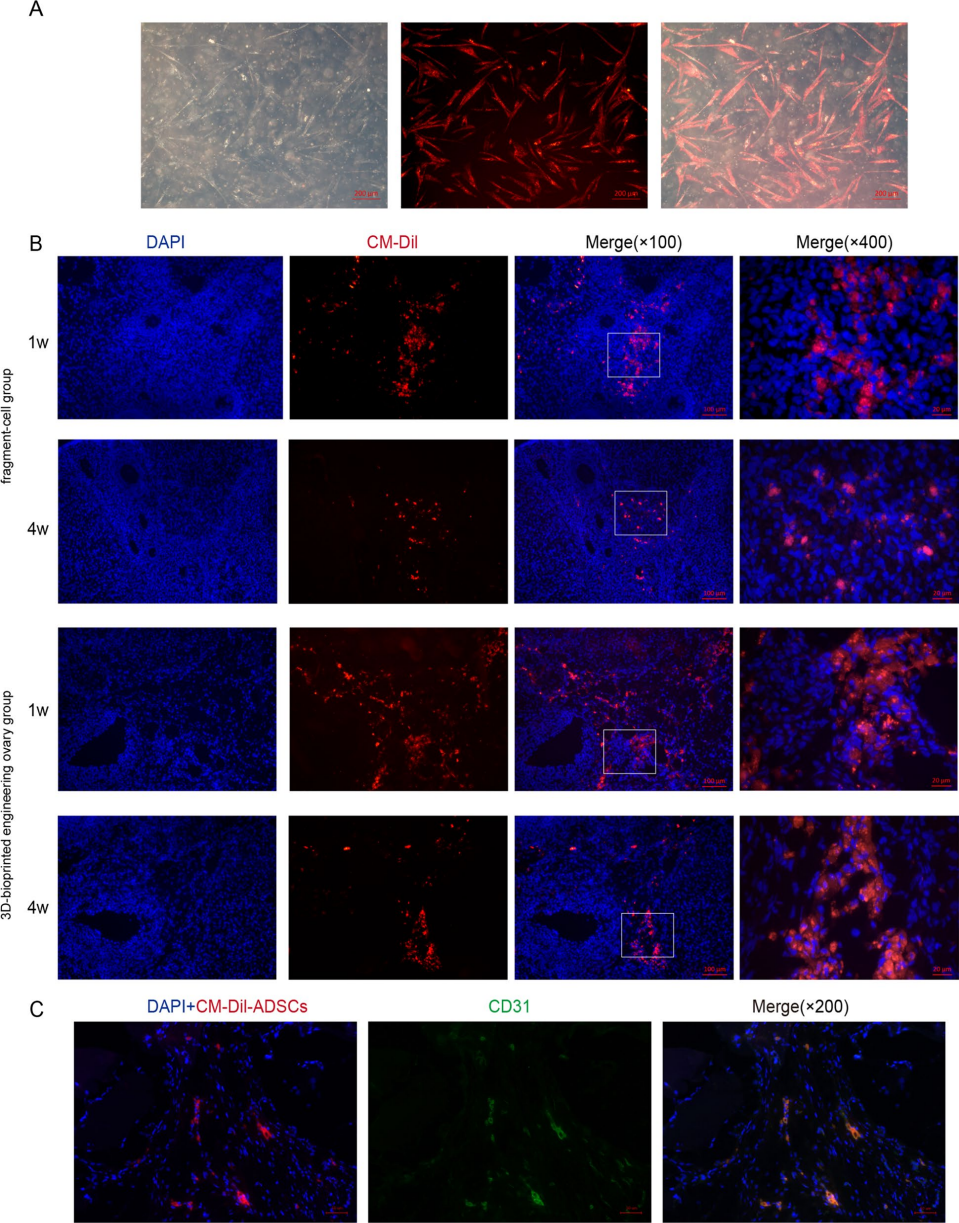
Localization and differentiation of ADSCs in grafts. **A** CM-Dil-stained ADSCs showed red fluorescence in vitro. Scale bar: 200 µm. **B** Localization of CM-Dil-stained ADSCs in the fragment-cell group and 3D-bioprinted engineering ovary group at 1 week and 4 weeks after transplantation. ADSCs were mainly distributed in interstitium of the ovary and progressively reduced over time, but the 3D-bioprinted engineering ovary group had higher retention ratio of ADSCs. Scale bar: 100 µm and 20 µm. **C** CM-Dil-stained ADSCs (red) were co-localized with CD31 staining (green) in vascular endothelial cells of 3D-bioprinted engineering ovary group at 4 weeks after transplantation. Scale bar: 50 µm

In the present study, we aimed to trace the in vivo localization of CM-Dil-labeled ADSCs with or without 3D printing scaffold after transplantation and the underlying mechanism of differentiation. After labeling, the cell membrane had a homogeneously distributed red fluorescence and did not show an obvious decline in cell passage (Fig. 1A). Furthermore, we traced the fate of CM-Dil-labeled ADSCs in the frozen section of grafts at 1 week and 4 weeks after transplantation. As shown in Fig. 1B, the red fluorescence signals were distributed in the interstitium of ovaries transplanted with CM-Dil-ADSCs or scaffold/ADSCs rather than in follicles. With time, the fluorescence signals progressively reduced either in ADSCs or in scaffold/ADSCs, but the higher retention ratio of scaffold/ADSCs was detected at 1 week and 4 weeks after transplantation. In addition, the positive staining for CD31 was green; the co-expressed site of red, blue, and green was white. We observed that several regions of the co-staining for CM-Dil and CD31 were in the area of vascular endothelial cells (Fig. 1C). These results indicated that 3D-printing dECM scaffold supported survival, growth, and proliferation of ADSCs and was conductive to retention of ADSCs and that a fraction of ADSCs might have differentiated into vascular endothelial cells to promote angiogenesis.

### Construction and transplantation of 3D-bioprinted engineering ovary

**Fig. 2:**
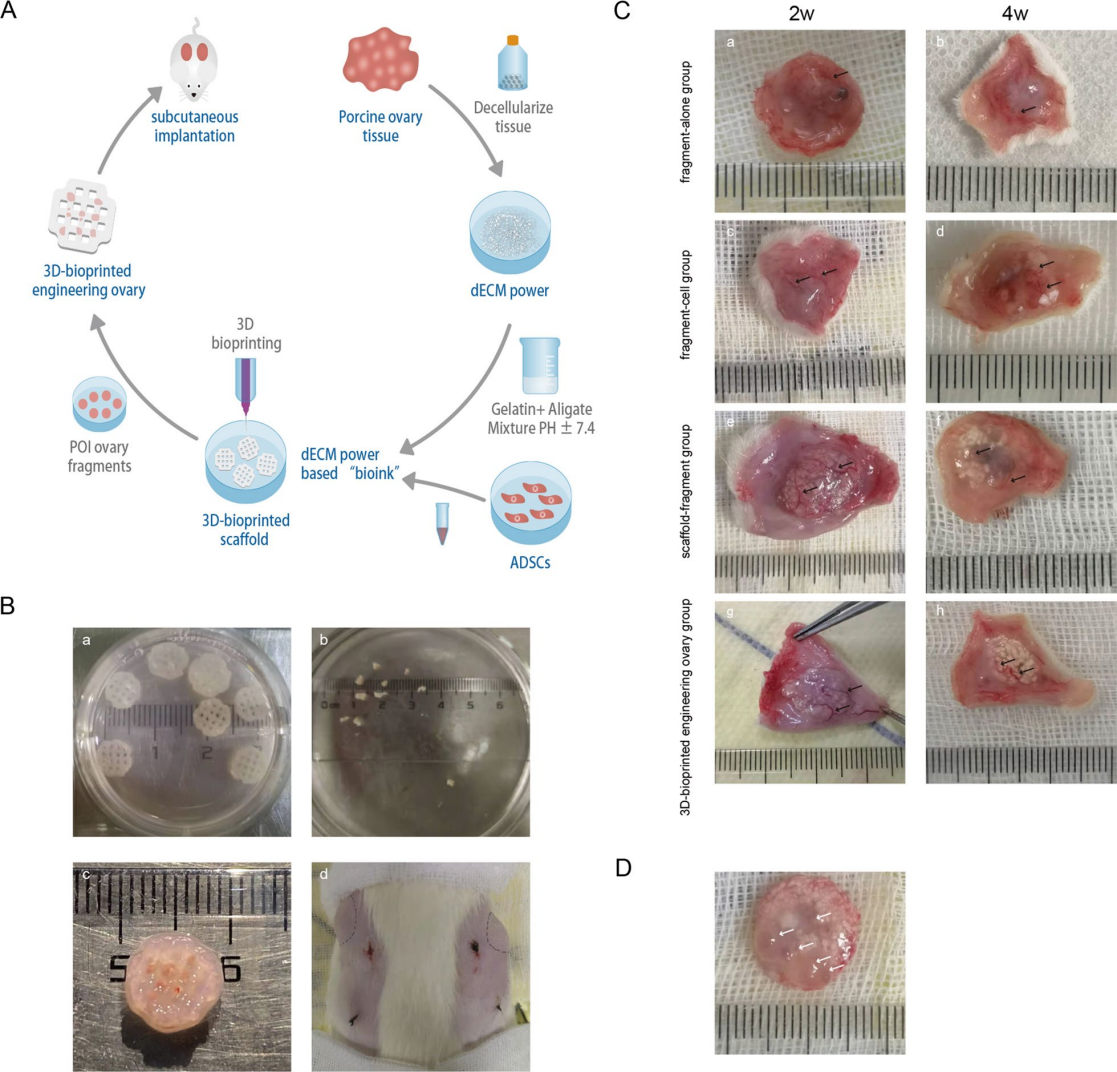
Profiles of grafts before and after transplantation. **A** Schematic representation of construction of 3D-bioprinted engineering ovary. **B** Photographs of 3D-bioprinted engineering ovary group related to surgical procedure, including preparing 3D scaffolds (**a**) and POI ovarian cortex fragments (**b**), wrapping ovarian fragments into 3D scaffolds (**c**) and subcutaneous transplantation (**d**). **C** Macroscopic evaluation of grafts at 2 and 4 weeks after transplantation. The ovarian fragments (with or without ADSCs) could be rejoined and wrapped by a hyaline membrane (**a**–**d**) and 3D constructs could maintain their structure (**e**–**h**). All grafts were surrounded by visible functional blood vessels (black arrow), but the 3D-bioprinted engineering ovary were more marked. **D** A large number of preovulatory follicles were observed in the 3D-bioprinted engineering ovary (white arrow)

The mechanical properties, 3D printability, biocompatibility and comparable cytocompatibility of dECM “bioink” had been demonstrated by our previous research [28]. The purpose of our study was to create a 3D-bioprinted engineering ovary. Thus, we queried whether the overall ovarian cortex fragments could be inserted while maintaining the 3D scaffold structure and integrity. Figure 2B shows that the fragments were totally inserted into the scaffold and the system remained polyporous and macroscopically intact. Grafts of each group were successfully transplanted into the subcutaneous pockets of the POI rats and all rats survived after surgery without any complications. All grafts were completely removed without infection, hematoma or wound dehiscence. At 2 and 4 weeks after transplantation, the constructs of ovarian fragments with/without ADSCs were found to be rejoined, integrated and wrapped by a hyaline membrane and adherent to the subcutis (Fig. 2C). The constructs of 3D scaffold combined with ovarian fragments (with or without ADSCs) could maintain their structure, but had a slight degradation with the prolongation of time (Fig. 2C). All grafts were surrounded by visible functional blood vessels, but grafts from the fragment-alone/3D scaffold group had less vascularization and more pale appearance than those from other two ADSC-containing groups. Moreover, macroscopically the 3D-bioprinted engineering ovary group had more surrounding blood vessels than other groups, and preovulatory follicles were found in the grafts (Fig. 2D).

### Analysis of ovarian morphology and follicle counting

**Fig. 3:**
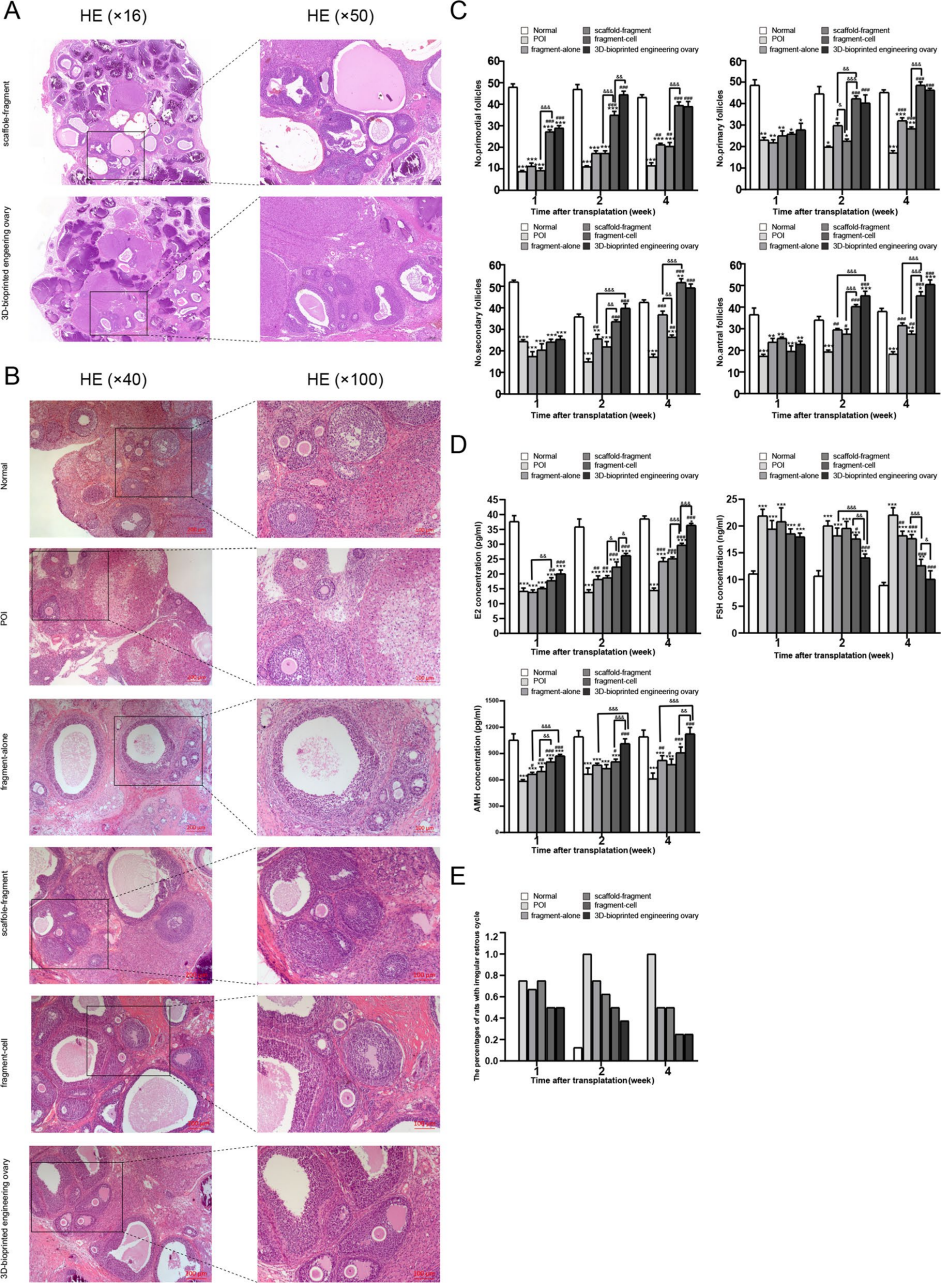
Ovarian functions after graft transplantation. **A** Histological analysis of biocompatibility between ovarian tissue and 3D scaffold with/without ADSCs. Scale bar: 500 µm and 200 µm. **B** Histological analysis of ovaries in six different groups. Scale bar: 200 µm and 100 µm. **C** Number of follicles at different stages counted at 1, 2, and 4 weeks after graft transplantation. **D** The levels of E2, FSH, and AMH were analyzed at 1, 2, and 4 weeks after graft transplantation. (* versus the normal group, # versus the POI group, & comparison between two groups; *, #, & *P* < 0.05; **, ##, && *P* < 0.01; ***, ###, &&& *P* < 0.001). **E** Changes of irregular estrous cycles

The initial experiments of our study were to investigate the biocompatibility between ovarian tissue and 3D scaffold, since the ambient suitable conditions were influencing factors for internal nutrient supply of ovarian tissue and follicle survival. We explored appropriate stent size and construction and found that 3D scaffold (8 × 8 × 3 mm^3^) could serve as proper microenvironment for survival of ovarian fragments. Figure 3A exhibits that any stage of healthy-looking follicles survived and developed within the dECM scaffold with/without ADSCs and did not provoke a marked pathological response.

Subsequent studies explored the therapeutic effects of ADSC-containing groups on the restoration of ovarian function. Histological staining of retrieved implants revealed that the number of follicles at any stage of development in POI group reduced markedly relative to normal group (Fig. 3B). In the fragment-alone group and scaffold-fragment group, the number of growing follicles increased, while there was no remarkable increase in primordial follicles (Fig. 3B). More excitingly, the pool of primordial follicles and growing follicles variously increased in fragment-cell group and 3D-bioprinted engineering ovary group (Fig. 3B).

We further quantified the number of follicles in different groups to evaluate the therapeutic effects (Fig. 3C). In the first two weeks after transplantation, although the number of primordial follicles and primary follicles in the fragment-alone group and scaffold-fragment group was not significantly different compared with the POI group, the number of secondary and antral follicles increased to some extent (*P* < 0.05). At four weeks after transplantation, the number of primordial follicles (*P* < 0.05), primary follicles (*P* < 0.05), secondary follicles (*P* < 0.05), and antral follicles (*P* < 0.05) in the above two groups were higher than that in the POI group, but still significantly lower than that in the ADSC-containing groups (*P* < 0.05). Based on comparisons of POI group and the other two treatment groups, there were markedly increased percentage of follicles at each stage observed from the first two weeks after transplantation in the fragment-cell group and 3D-bioprinted engineering ovary group (*P* < 0.05) and reached normal or near-normal range after four weeks (*P* > 0.05), but no significant difference was reported between the two groups (*P* > 0.05). Overall, these data indicated the effects of ovarian fragmentation combined with ADSCs on the increase in ovarian reserve and reduction in ovarian injury in the POI rats.

### Estrous cycle and hormone levels

We detected the estrous cycle and serum hormone levels of E2, AMH, and FSH to investigate the therapeutic effects of grafts transplantation on restoration of ovarian function in POI rats. Our finding demonstrated that the proportion of irregular estrous cycles after chemotherapy was decreased from the second week in the four treatment groups. At the end of the fourth week, 75% (3/4) of rats in fragment-cell group and 3D-bioprinted engineering ovary group had regular estrous cycle, which was significantly higher compared to POI group and the other two treatment groups (Fig. 3E). The ELISA results indicated that the levels of FSH and AMH were slightly recovered in the four treatment groups relative to the POI group starting with the first week, especially the fragment-cell group and 3D-bioprinted engineering ovary group. By contrast, the recovery of E2 started in the second week after transplantation and gradually increased with prolonged recovery. However, at the end of the fourth week, the levels of E2 and AMH in 3D-bioprinted engineering ovary group were significantly higher than those in other treatment groups and at near-normal level (Fig. 3D, *P* < 0.05). In addition, the level of FSH was decreased to the normal level at four weeks after treatment in 3D-bioprinted engineering ovary group and differed markedly from the other treatment groups (Fig. 3D, *P* < 0.05). These findings confirmed that all of the treatment groups could upgrade serum hormone levels and restore ovarian function, which was most pronounced in fragment-cell group and 3D-bioprinted engineering ovary group, especially the latter one.

### Proliferation and angiogenesis

**Fig. 4:**
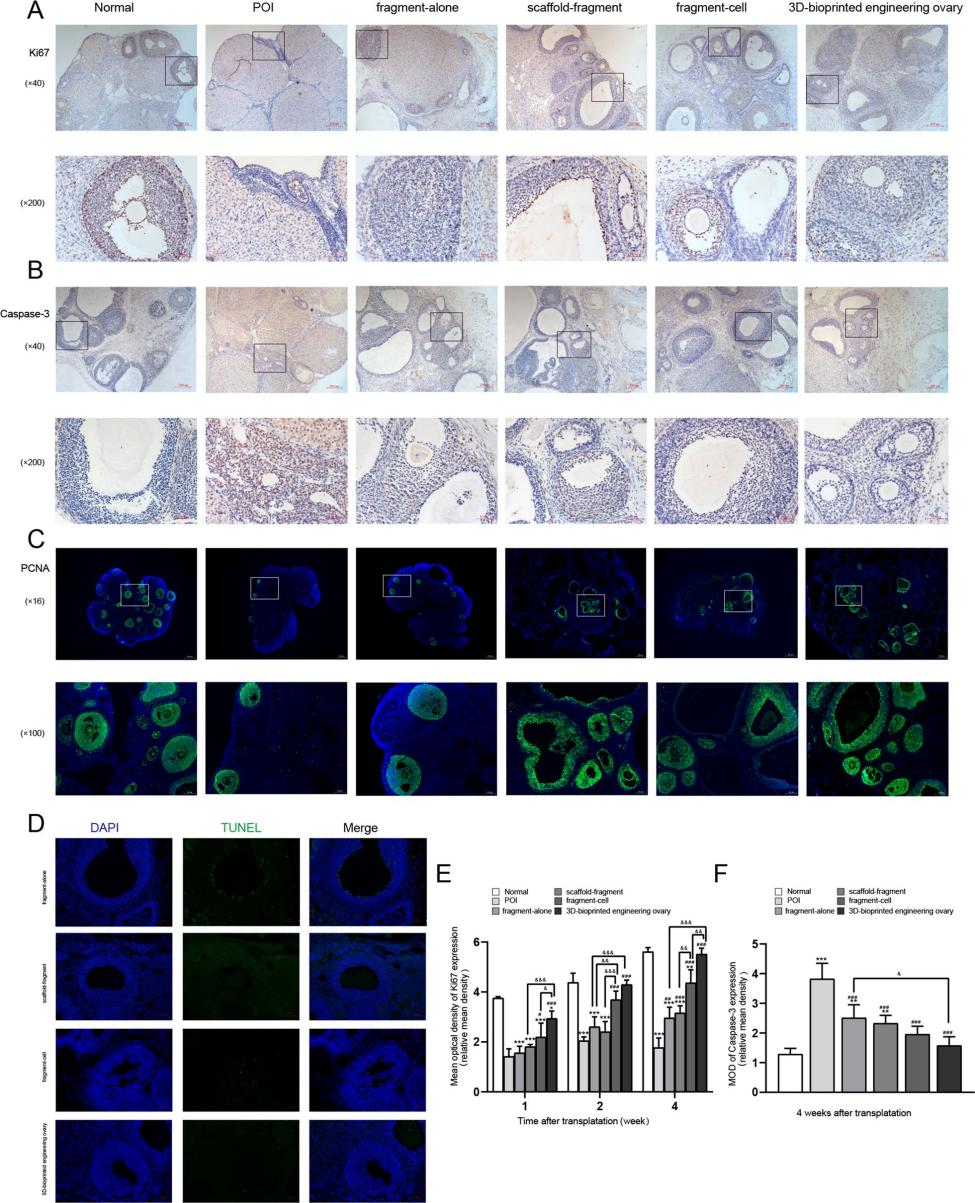
Proliferation and apoptosis of granulosa cells (GCs) after graft transplantation. Representative immunohistochemical images of Ki67 (**A**) and caspase-3 (**B**) in six different groups. Scale bar: 50 µm and 200 µm. **C** Representative immunofluorescence staining of PCNA in six different groups. Scale bar: 500um and 100 µm. **D** Representative pictures of TUNEL staining from the four treatment groups. Green staining represents apoptotic GCs. Scale bar: 50um. The mean optical density (MOD) of ki67-positive (**E**) and caspase-3-positive areas (**F**) were analyzed by Image-Pro Plus. (* versus the normal group, # versus the POI group, & comparison between two groups; *, #, & *P* < 0.05; **, ##, && *P* < 0.01; ***, ###, &&& *P* < 0.001)

Ki67 and PCNA (molecular proliferation marker) were investigated, and caspase-3 and TUNEL staining (molecular apoptosis marker) were performed to further estimate the proliferation and apoptosis of GCs of follicles at different stages in the grafted tissues. As shown in our results, there were more Ki67-positive follicles in the fragment-cell group and 3D-bioprinted engineering ovary group compared with other treatment groups at 1, 2, and 4 weeks after transplantation, especially the 3D-bioprinted engineering ovary group at 4 weeks after transplantation, which was similar to that of the normal group (Fig. 4A/E, *P* < 0.05). Similarly, our results indicated that PCNA showed more positive proliferative GCs in the fragment-cell group and 3D-bioprinted engineering ovary group, which were higher than those in the other groups by IF staining (Fig. 4C). Furthermore, we evaluated the apoptosis of follicles in different groups using caspase-3 and TUNEL staining at 4 weeks after transplantation. The caspase-3 immunostaining showed that the apoptosis of follicles decreased significantly in four treatment groups—fragment-alone group, scaffold-fragment group, the fragment-cell group, and 3D-bioprinted engineering ovary group—compared to the POI group, and that the disparities were most pronounced in 3D-bioprinted engineering ovary group (Fig. 4B/F, *P* < 0.05). The same result was also confirmed by the TUNEL staining (Fig. 4D).

**Fig. 5:**
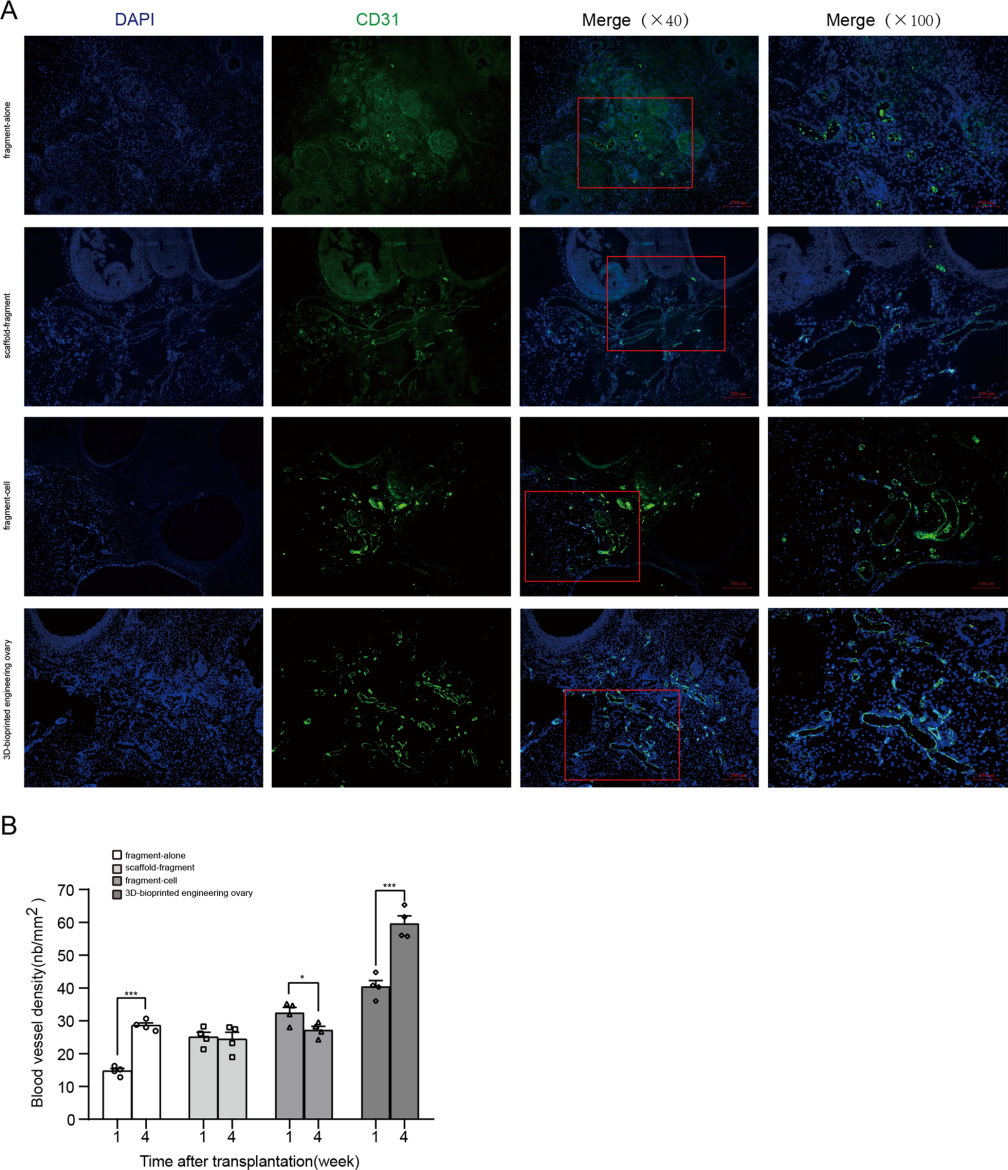
Assessment of graft revascularization. **A** Angiogenesis shown in representative images of CD31-positive vessels in fragment-alone group, scaffold-fragment group, fragment-cell group, 3D-bioprinted engineering ovary group at 1 week after graft transplantation. Scale bar: 200 µm and 100 µm. **B** Data analysis of blood vessels density (number of blood vessels (nb) per mm.^2^) was performed in four treatment groups at 1 week and 4 weeks after transplantation. The significantly higher vessels density in 3D-bioprinted engineering ovary group from 1 to 4 weeks after transplantation indicated the more progressed and continuous vascular remodeling (* *P* < 0.05, ** *P* < 0.01, *** *P* < 0.001)

Our above results had confirmed more retention of ADSCs in the 3D-bioprinted engineering ovary group. Therefore, we further evaluated whether more ADSCs encapsulation affected the incidence and degree of local dECM neovascularization, which had direct effects on the follicle survival and longevity of the grafts. By IF analysis, we detected higher CD31-positive signals and functioning blood vessels in the 3D-bioprinted engineering ovary group, as compared with the other groups (Fig. 5A). Additionally, a larger number of positively stained cells for CD31 was found at 1 week after transplantation and also increased significantly at 4 weeks, indicating the continuous vascular remodeling (Fig. 5B, *P* < 0.05). In the fragment-cell group, although there was neovascular infiltration at 1 week after transplantation, the vascularization was not maintained but significantly decreased at 4 weeks after transplantation (*P* < 0.05). In the fragment-alone group and scaffold-fragment group, we also detected neovascularization at 1 week and 4 weeks after transplantation, but the number of vessels was significantly less than that in the other two groups (*P* < 0.05).

### Activation of the PI3K/AKT pathway to promote angiogenesis

**Fig. 6:**
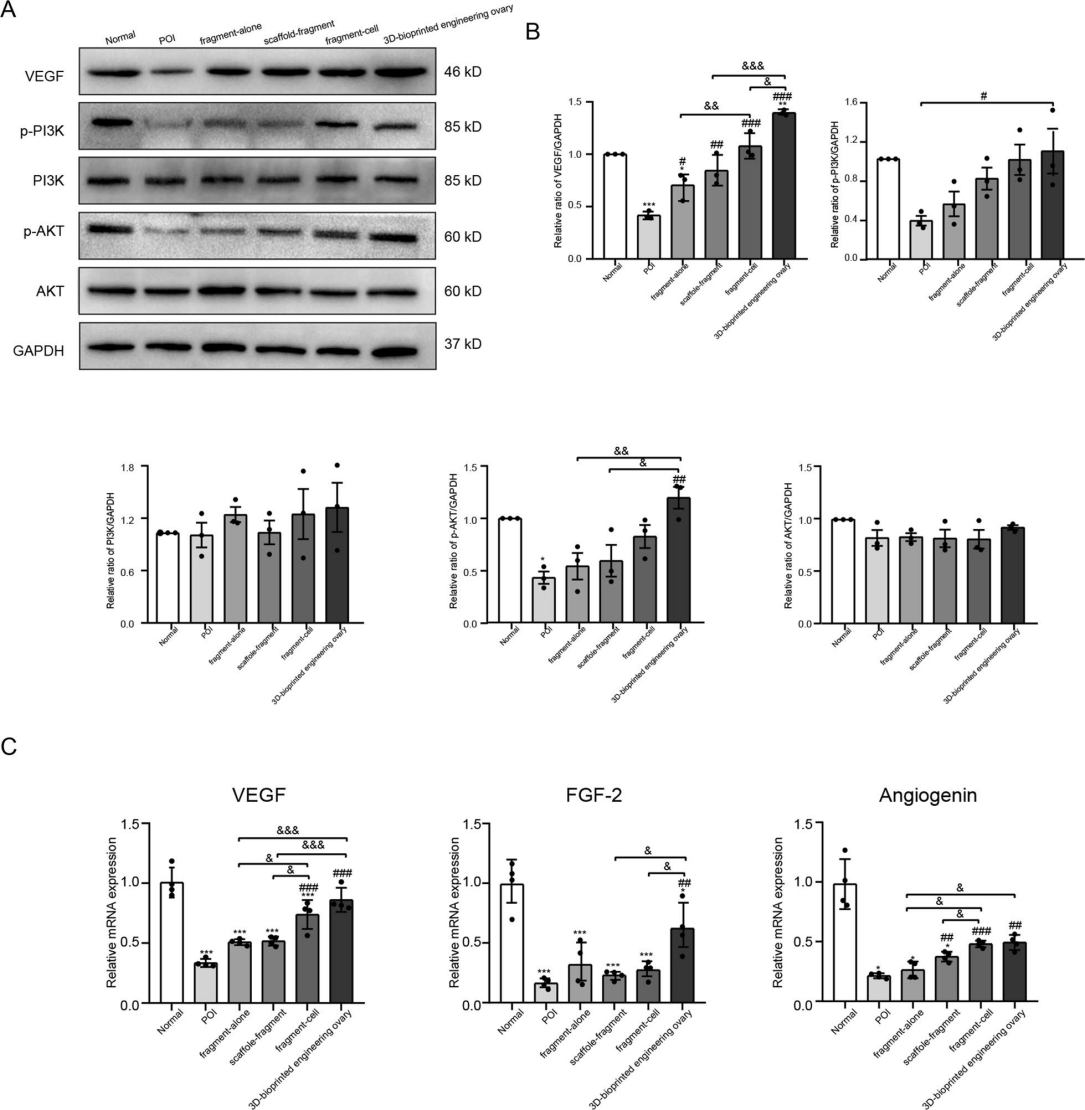
Promoted angiogenesis by regulating PI3K/AKT pathway and secreting proangiogenic factors. **A** Representative VEGF, p-PI3K, PI3K, p-AKT and AKT bands of PI3K/AKT pathway using GAPDH as the internal reference. **B** Quantitative analysis of protein expression showed that the expression level of p-PI3K, p-AKT and VEGF was increased in fragment-cell group and 3D-bioprinted engineering ovary group, while the level of PI3K and AKT did not show significant difference in different groups. **C** The mRNA levels of VEGF, FGF-2, and angiogenin were upregulated in the 3D-bioprinted engineering ovary group. (* versus the normal group, # versus the POI group, & comparison between two groups; *, #, & *P* < 0.05; **, ##, && *P* < 0.01; ***, ###, &&& *P* < 0.001)

To gain further understanding of the mechanism of ADSC-containing groups promoting the angiogenesis of the grafts, the expression of proangiogenesis-related proteins related to PI3K/AKT signal pathway was detected. Compared with POI group, higher p-AKT, p-PI3K expression and VEGF upregulation were detected in the fragment-cell group and 3D-bioprinted engineering ovary group (Fig. 6A/B, *P* < 0.05), while the levels of total AKT and PI3K did not significantly differ from the other two groups (Fig. 6A/B, *P* > 0.05). Moreover, although the expression levels of the above proteins in 3D-bioprinted engineering ovary group were higher than those in the fragment-cell group, there was no significant difference in the expression of p-AKT and p-PI3K (Fig. 6A/B, *P* > 0.05) except VEGF (Fig. 6A/B, *P* < 0.05). As recognized by the mRNA expression of VEGF, FGF-2 and angiogenin, there was a significant upregulation in ADSC-containing groups compared with the POI group and the other two treatment groups, especially the 3D-bioprinted engineering ovary group (Fig. 6C, *P* < 0.05). Taken together, these findings suggested the role of ADSCs in promoting angiogenesis possibly by activating the PI3K/AKT pathway.

## Discussion

In our study, we designed for the first time a novel 3D-bioprinted engineering ovary, which combined ovarian fragments with 3D printing scaffold employing dECM-derived “bioink” containing ADSCs, to restore impaired ovarian function in POI. The results of our study demonstrated that 3D scaffold not only prolonged the retention of ADSCs, but also increased blood flow at one week after transplantation accompanied by the prolonged time of blood perfusion, thereby decreasing hypoxia–ischemia after transplantation and improving follicle survival. Next, our study further demonstrated that the 3D-bioprinted engineering ovary, compared with other treatment groups, increased production of follicles at various stages and showed a significantly faster recovery of hormone levels and estrous cycle, increased proliferation, and reduced apoptosis of ovarian cells. Finally, we substantiated that our therapeutic mechanism of POI involved facilitating angiogenesis by regulating the PI3K/AKT pathway.

With serious implications in women’s physical and psychological well-being as well as quality of life, the management of POI is gaining increasing attention. Currently, first-line hormone replacement therapy (HRT) only ameliorates its symptoms, but has non-ideal therapeutic effects due to various side reactions, non-fundamental improvement of ovarian function and inability to activate primordial follicles [34]. Egg donation has been generally used to women who have fertility demand [1], but the limited success and inability to own their genetic offspring urgently require new therapeutic approaches to reverse this situation [35]. In recent years, IVA is a rapidly advancing method of activating and regulating follicle growth developed by Kawamura et al. [3] and Suzuki et al. [10]. This approach has been successfully applied to patients with POI, which enabled them to conceive with their own genome. The conventional IVA is a two-step process that first requires in vitro culture of ovaries to activate dormant primordial follicles, followed by the second step of ovarian cortical fragmentation to activate follicle growth [2, 3]. However, this technique has been hampered by secondary surgical trauma, low rate of pregnancy and potential carcinogenic effect of pharmacologic substances [4, 5]. Thus, a less invasive method of drug-free IVA has been developed to promote follicle growth by disrupting Hippo signaling alone (only one surgery). Drug-free IVA is a recent technique that has been applied to patients with POI, diminished ovarian reserve (DOR) and poor ovarian response (POR) [6–9]. The effectiveness of drug-free IVA has been confirmed by the rate of clinical pregnancy and birth rate. Compared with conventional IVA, a number of researchers have suggested that the drug-free IVA has higher pregnancy rate, birth rate, and the number of collected oocytes [13]. To date, despite several success pregnancies and live birth that have been reported from the drug-free IVA, various issues have greatly restricted its application, such as the loss of substantial number of primordial follicles, unimproved quality of age-associated oocyte, and poor therapeutic success [2, 13].

It is well known that the number of residual follicles and disease duration are the major determinant of therapeutic success of IVA [11], and ischemic injury early after transplantation is one of the major negative issues affecting the survival of follicles [36]. Therefore, we should attempt to improve ovarian reserve and promote early revascularization to improve follicle survival [37, 38]. A number of studies have reported the angiogenic potential of ADSCs [40] and pre-constructed a prepared vascularized grafting region via ADSCs to promote ovarian cell survival [41, 42]. For the above-stated reasons, we combined the two therapeutic methods and observed that the fragment-cell group had early revascularization of grafts, restoration of hormone levels, and continuously increasing proportion of growing follicles as well as a high percentage of primordial follicles during the 4 weeks after transplantation. This demonstrated a protective effect of ADSCs on ovarian reserve and follicular activation of ovarian fragmentation, which was considered to be responsible for more rapid recovery of ovarian function. However, we traced the distribution of CM-Dil-labeled ADSCs and found that the fluorescence signals significantly reduced with prolonged time after transplantation. Additionally, although we observed the revascularization of grafts within 1 week, the vascularization was not maintained but significantly decreased at 4 weeks. The therapeutic effects were diminished due to their rapid diffusion [22, 43] and low viability of retained cells in the target tissue [44].

Recently, the application of biomaterials for regenerative medicine, such as collagen, hydrogel, and fibrin, is a promising approach to delivery and maintenance of seeding cells in the target organ [21, 45–47]. However, these biomaterials are less able to fully simulate the complex extracellular ecological environment for survival of various cells [48]. Thus, enormous endeavors have been devoted to the application of dECM, since they support a variety of cells due to their complex tissue-specific properties and unique composition of functional components [49, 50], thus providing excellent biochemical functionality and biocompatibility [51] for tissue remodeling and function recovering [33, 52]. Our previous studies demonstrated that our dECM had maximized removal of cells while preserving the structure and composition of native tissue [27, 28]. In addition, we had also successfully designed a 3D printing scaffold employing dECM “bioink” encapsulating bone marrow mesenchymal stem cells (BMSCs), which demonstrated a promising approach to vagina reconstruction [28]. To our knowledge, the obtained 3D scaffold recreated in vitro the complexity of in vivo native tissue milieu, overcoming the disadvantages of 2D culture system (i.e., loss of tissue-specific architecture and changes in cellular morphology and function) and providing the structural support between the cell culture environment and the surrounding tissue environment [25]. In addition, the 3D scaffold not only improved cell retention, proliferation, and differentiation, but also promoted angiogenesis, nutrient supply, and functional recovery by loading more cells [27, 28]. Therefore, we engineered a novel 3D-bioprinted engineering ovary composed of 3D scaffold containing ADSCs and ovarian cortical fragments. Our results confirmed that 3D scaffold could provide ADSCs with an appropriate niche and thus increased the retention and survival of ADSCs during a long period, while the ADSCs alone experienced significant cell loss within 4 weeks after transplantation (Fig. 1B). With more loading of ADSCs into 3D scaffold, accompanied by an increased secretion of soluble growth factors (VEGF, FGF2, and angiogenin) [21], the number of blood vessels was significantly increased as compared with other groups. In addition, the grafted ADSCs could gain vascular endothelial-like phenotypes (Fig. 1C), which was in agreement with our previous research [28, 53], thereby further explaining why the 3D-bioprinted engineering ovary had more pronounced vascularization. Next, we further evaluated the effects of the novel 3D-bioprinted engineering ovary on restoration of ovarian function. We observed that the proportion of follicles at all development stages continued to increase significantly rather than only partial activation of growing follicles, which was considered to be associated with the early revascularization after transplantation and the ADSCs’ capacity of selective activation of primordial follicles and in turn improved protection of ovarian reserve. We also observed obviously increased ki67 staining and decreased caspase-3 staining of GCs after 3D-bioprinted engineering ovary transplantation, indicating that more retention ADSCs had better protective effects of GCs induced by CTX. Additionally, we examined the restoration of hormone levels and estrous cycle, which were other evaluation indexes for recovery of ovarian function. Taken together, these results verified that 3D-bioprinted engineering ovary transplantation resulted in better ovarian functional recovery than other treatment groups, which might provide a novel therapeutic strategy for POI patients.

Our above studies demonstrated that we successfully reconstructed a 3D-bioprinted engineering ovary by remodeling of an early and long-term vascular system in the construct, which increased the survival of the follicles and ovarian function. To further elucidate the mechanism responsible for angiogenesis, we detected the mRNA expression levels of angiogenic factors (e.g., VEGF, FGF-2, and angiogenin) [54–56] in all groups. It was found that their mRNA expression levels in 3D-bioprinted engineering ovary were significantly higher than those in other groups, suggesting that the presence of rich vascular network in the construct was attributed to a high concentration of pro-angiogenic factors induced by ADSCs. It is generally accepted that PI3K/AKT signaling pathway plays a crucial role in stimulating angiogenesis, activating primary follicles, and further reducing ovarian damage [39, 57, 58]. Our results revealed elevated p-PI3K, p-AKT, and VEGF levels in the fragment-cell group and 3D-bioprinted engineering ovary group, especially the latter one, suggesting that the activation of PI3K/AKT signaling pathway might be involved in the regulation of ADSCs’ effects on angiogenesis. Taken together, these studies indicated that transplantation of 3D-bioprinted engineering ovary promoted angiogenesis and restored ovarian functions possibly through the PI3K/AKT signaling pathway.

The 3D-bioprinted engineering ovary developed in this study is a critical first step to evaluate the effectiveness of exploring such a method for restoring ovarian function in POI. Our further study will focus on improvement of 3D-bioprinted scaffold for orthotropic ovary transplantation, so as to evaluate the efficiency of fertility improvement and further explore its mechanism of promoting angiogenesis. In this study, we investigated the effectiveness of ADSCs in protecting ovarian reserve and regulating angiogenesis, but failed to ascertain whether this method could improve quality of oocytes. Therefore, a series of relevant experiments will be performed in our follow-up studies. In our study, we successful removed grafts and found excellent biocompatibility between scaffold and ovarian tissues; however, inflammation-based indicators in serum and grafts were not detected in our study, which need further additional exploration. With preliminary study presented here, more animals and extended observation time are needed in our future studies to provide sufficient supporting evidence for future clinical studies.

Excitingly, 3D-bioprinted engineering ovary transplantation is indeed showing great potential for restoring impaired ovarian function in POI. Our results are encouraging, although clinical applications still have a long way to go. Our studies clearly demonstrated that 3D-bioprinted ADSCs-loaded scaffold constructed a higher rate of vascularization and reduced massive follicle loss in the early grafting period, which could compensate for the disadvantages of IVA to some extent. Moreover, our findings raise the possibility that the 3D-bioprinted ADSCs-loaded scaffold may provide an effective method for grafting of cryopreserved ovarian tissue.

## Conclusion

In conclusion, we have demonstrated that 3D-bioprinted scaffold could improve retention of ADSCs and revascularization in the grafts. In addition, to our knowledge, we designed for the first time that a 3D-bioprinted engineering ovary composed of drug-free IVA and ADSCs, which contributed to a more effective approach to restoration of ovarian function in POI rats.

## Supplementary Information

**Additional file 1: Fig. S1** Characterization and identification of ADSCs. (A) Morphological features of ADSCs at the primary passage and third passage. Scale bar: 100um. (B) Flow cytometric analysis of fifth- passage ADSCs showed that cells were positive for CD29, CD73, CD105, CD44, CD90 expression and negative for CD45 and HLA-DR expression. (C) The multi-potent differentiation capacity of ADSCs was evaluated by osteogenic and adipogenic induction. Scale bar: 100 µm.

**Additional file 2: Fig. S2** Decellularization of porcine ovarian tissues and evaluation of the POI. (A) HE staining showed visible follicles and nuclei in the native ovary tissue while no visible nuclear staining in the decellularized extracellular matrices (dECMs), but leaving intact pink-staining extracellular matrix. DAPI staining detected that no blue nuclei remained in the dECMs. Masson staining revealed that intact collage preserved after the decellularization process. Scale bar: 200 µm. (B) HE staining of the ovary in normal and POI group. Scale bar: 100 µm and 200 µm. (C) TUNEL staining was used to detect the apoptosis of granulosa cells (GCs) in the normal and POI group. Positive apoptotic GCs stained green with TUNEL-FITC while nuclei were stained blue by DAPI. Scale bar: 50 µm. (D) Changes in rat body weight after CTX injection and graft transplantation. The dotted line represents the time of graft transplantation. (E) Representative estrous cycles of two rats in normal group and POI group. P: proestrus; E: estrus; M: metestrus; D: diestrus (a). The average estrous cycle length in normal and POI groups (b). (F) E2 level and FSH level were examined by ELISA after CTX injection for 14 d (* *P* < 0.05, ** *P* < 0.01, *** *P* < 0.001).

## Data Availability

All data generated or analyzed during this study are included in this published article and its Additional files 1 and 2.

## Abbreviations

Drug-free IVA: Drug-free in vitro activation
POI: Premature ovarian insufficiency
MSCs: Mesenchymal stem cells
ADSCs: Adipose-derived stem cells
PTEN: Phosphatase and tensin homolog
PI3K: Phosphatidylinositol-3-kinase
AKT: Protein kinase B
FOXO3: Forkhead box O3
GCs: Granulosa cells
dECM: Decellularized extracellular matrix
FCM: Flow cytometry
HE: Hematoxylin and eosin
DAPI: 4′6-Diamidino-2-Phenylindole
PBS: Phosphate-buffered saline
CTX: Cyclophosphamide
OCT: Optimal cutting temperature
IF: Immunofluorescence
ELISA: Enzyme-linked immunosorbent assay
E2: Estradiol
FSH: Follicle-stimulating hormone
AMH: Anti-Müllerian hormone
BSA: Bovine serum albumin
cDNA: Complementary DNA
HRT: Hormone replacement therapy
DOR: Diminished ovarian reserve
POR: Poor ovarian response
BMSCs: Bone marrow mesenchymal stem cells
