# Supplementary figures and images for: Drug-free in vitro activation combined with 3D-bioprinted adipose-derived stem cells restores ovarian function of rats with premature ovarian insufficiency
Source: Stem Cell Res Ther. 2022 Jul 26;13:347. doi: 10.1186/s13287-022-03035-3 (PMC9327214; doi:10.1186/s13287-022-03035-3)

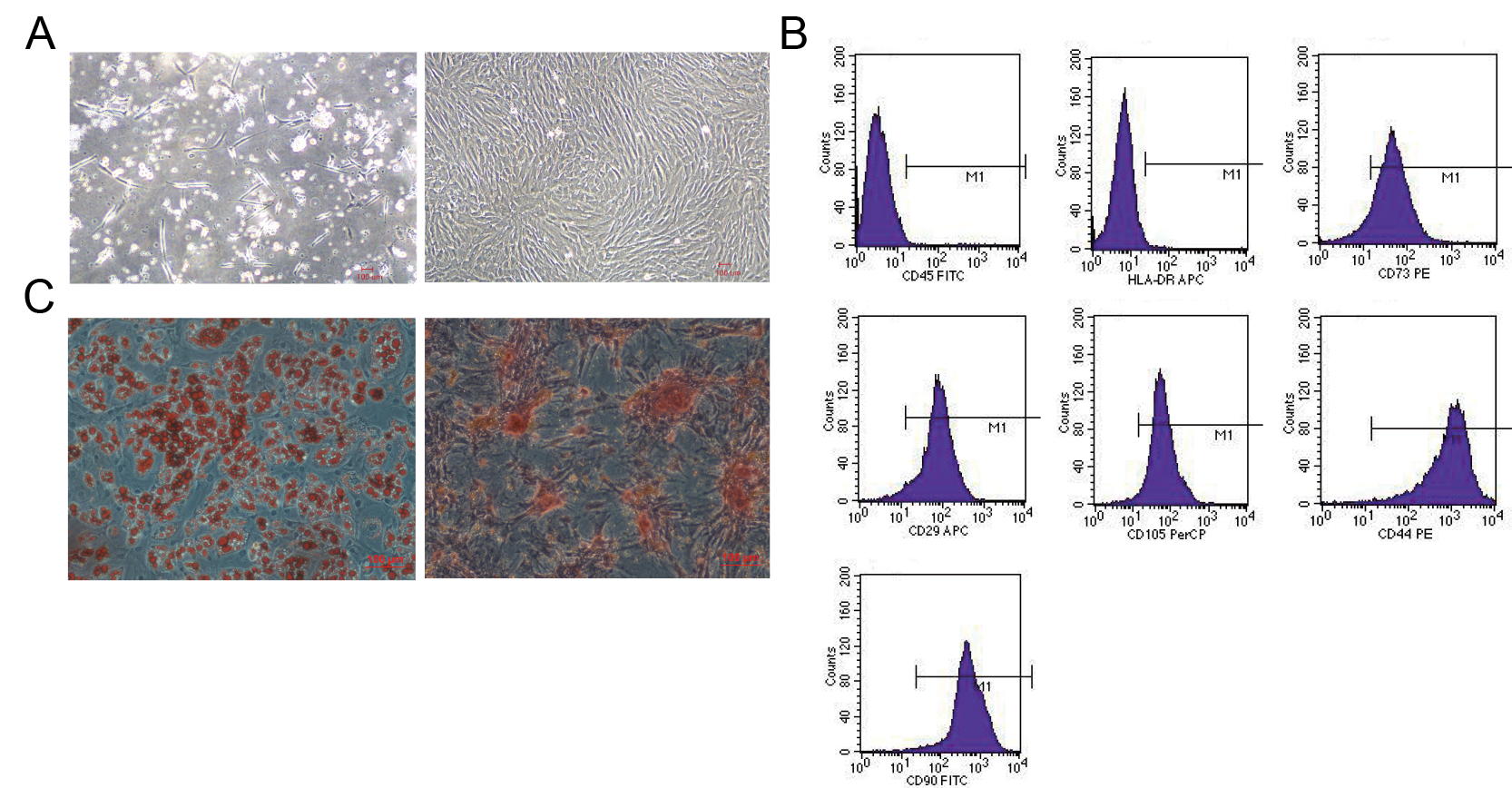

Supplement: Supplementary file 1 — Additional file 1: Fig. S1 Characterization and identification of ADSCs. (A) Morphological features of ADSCs at the primary passage and third passage. Scale bar: 100um. (B) Flow cytometric analysis of fifth- passage ADSCs showed that cells were positive for CD29, CD73, CD105, CD44, CD90 expression and negative for CD45 and HLA-DR expression. (C) The multi-potent differentiation capacity of ADSCs was evaluated by osteogenic and adipogenic induction. Scale bar: 100 µm. [file 13287_2022_3035_MOESM1_ESM.tif]

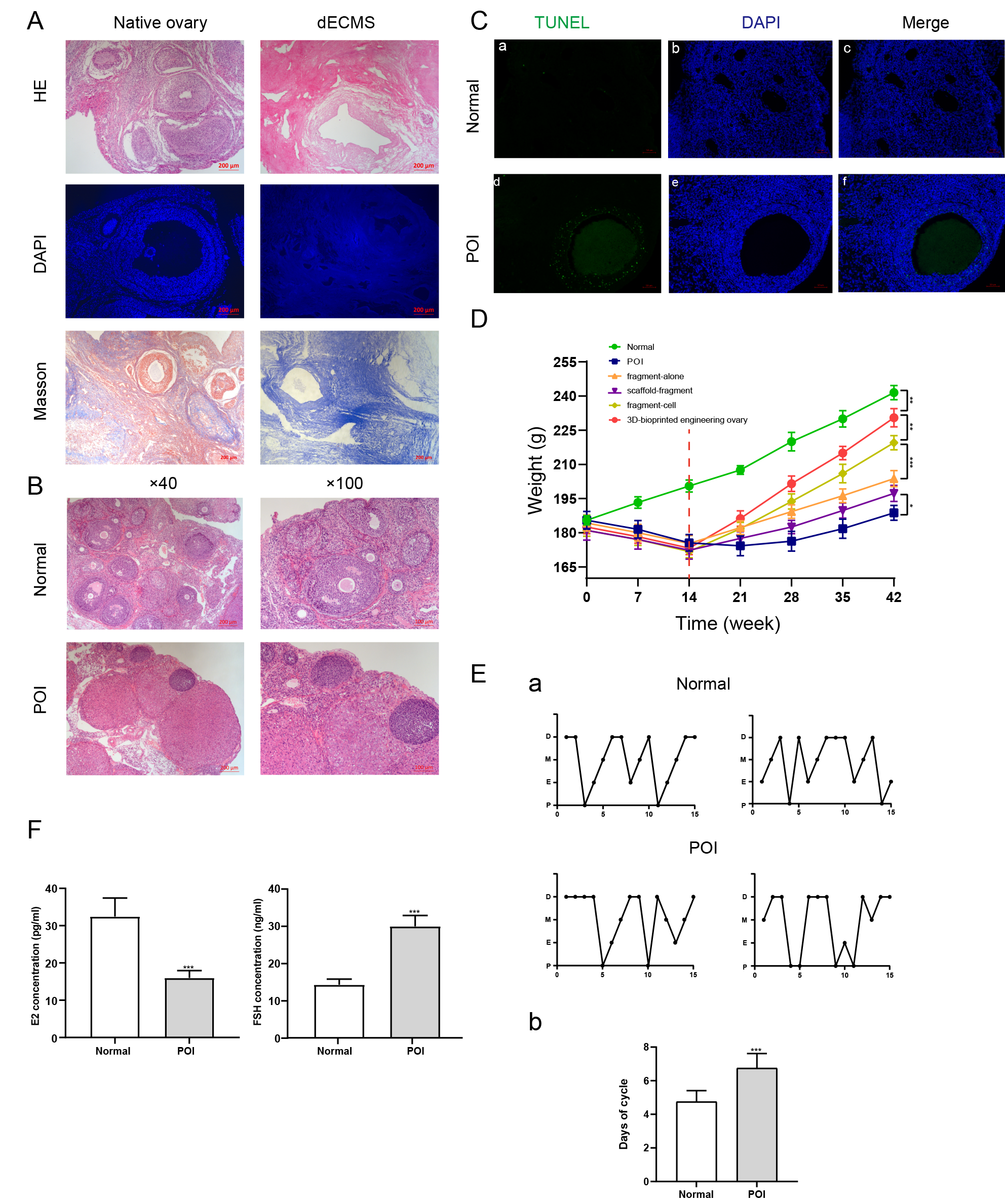

Supplement: Supplementary file 2 — Additional file 2: Fig. S2 Decellularization of porcine ovarian tissues and evaluation of the POI. (A) HE staining showed visible follicles and nuclei in the native ovary tissue while no visible nuclear staining in the decellularized extracellular matrices (dECMs), but leaving intact pink-staining extracellular matrix. DAPI staining detected that no blue nuclei remained in the dECMs. Masson staining revealed that intact collage preserved after the decellularization process. Scale bar: 200 µm. (B) HE staining of the ovary in normal and POI group. Scale bar: 100 µm and 200 µm. (C) TUNEL staining was used to detect the apoptosis of granulosa cells (GCs) in the normal and POI group. Positive apoptotic GCs stained green with TUNEL-FITC while nuclei were stained blue by DAPI. Scale bar: 50 µm. (D) Changes in rat body weight after CTX injection and graft transplantation. The dotted line represents the time of graft transplantation. (E) Representative estrous cycles of two rats in normal group and POI group. P: proestrus; E: estrus; M: metestrus; D: diestrus (a). The average estrous cycle length in normal and POI groups (b). (F) E2 level and FSH level were examined by ELISA after CTX injection for 14 d (* P < 0.05, ** P < 0.01, *** P < 0.001). [file 13287_2022_3035_MOESM2_ESM.tif]
